# Portable Non-Destructive Magnetic Resonance Sensor for Assessing the Aging Status of Silicon Rubber Insulators

**DOI:** 10.3390/nano12213847

**Published:** 2022-10-31

**Authors:** Pan Guo, Liling Wang, Rui Wang, Bing Li, Zhirui Zhao

**Affiliations:** 1 College of Physics and Electronic Engineering, Chongqing Normal University, Chongqing 401331, China; 2 Urumqi Power Supply Company, State Grid Xinjiang Electric Power Co., Ltd., Urumqi 830063, China

**Keywords:** silicone rubber insulators (SRIs), nuclear magnetic resonance (NMR), aging detection, portable sensor, non-destructive detection

## Abstract

Silicone rubber insulators (SRIs) are widely used in high-voltage power grids. Due to high-voltage fields and harsh environmental conditions, SRIs eventually deteriorate with use in the power grid, decreasing their insulating performance and operational life and contributing to transmission line failures. Therefore, quantitatively assessing the aging status of SRIs is crucial. In this study, we evaluated the viability of the magnetic resonance method for assessing the age of SRIs at the level of chemical structure; we built and made a portable magnetic resonance sensor, and evaluated the sensor’s functionality. By measuring the SRI sheds at various service times, it was discovered that the equivalent transverse relaxation time, *T*_2*eff*_, can describe the degree of aging of the SRIs. The results of the magnetic resonance measurements were also compared with those of the static contact angle method, and the two measurement methods yielded the same conclusions. However, the magnetic resonance method was more sensitive than the one using the static contact angle method.

## 1. Introduction

Insulators play an important role in the electrical insulation and mechanical stress of high-voltage power transmission lines. The insulator is composed of three parts: the shed, the core rod, and the metal fittings. The shed is often made of silicone rubber, glass, or ceramic. Due to their superior electrical and mechanical qualities, as well as resistance to fouling, SRI materials have largely replaced other insulation materials in power grids [1,2,3]. SRI materials in long-term outdoor operations are subject to strong ultraviolet light, strong electric fields, fouling, and other factors, which will inevitably lead to serious aging phenomena such as chalking, hardening, hydrophobic deterioration, and other aging phenomena. These aging phenomena lead to transmission line failures, and threaten the safety of power systems [4,5,6,7]. Therefore, a quantitative analysis of the aging status and aging rule of SRI materials is extremely important.

At present, the detection methods for the aging of the SRI shed at home and abroad mainly include the direct observation method, the HC (hydrophobicity class) classification method [8,9,10,11,12], the contact angle method [11,13], the leakage current method [14], the thermally stimulated current method [13,15,16], Fourier transform infrared spectrometry [17,18], and so on. The direct observation method and the HC classification method are directly observed with the naked eye, but their accuracy is poor and they are subject to several inaccuracies, due to the O&M staff’s subjective judgments. The leakage current method estimates the operating condition of SRIs by measuring the leakage current. The measurement of leakage current may show in real time how several dynamic factors, such as voltage, temperature, and fouling, affect the operating conditions of SRIs [14,19], but this method is limited by the randomness of the measurement and the uncertainty of the measuring standard. The contact angle method, the thermally stimulated current method and Fourier transform infrared spectrometry could be interfered with by environmental elements, when measuring. Generally, these measurements can only be carried out under laboratory conditions. In addition, these are destructive testing methods for the SRI sheds that need to be cut.

The power engineers’ ideal method for determining the SRIs’ aging condition should be quick, easy, and inexpensive to implement. It should also be able to assess the silicone rubber’s aging status at the microscopic level and be widely applicable as an engineering measurement method. Due to its quick, practical, and quantitative nondestructive measurements, magnetic resonance technology has been used in materials science to measure the crosslink density of polymeric materials, regulate the quality of rubber production processes, and detect the aging of rubber and polymer materials [20,21]. Commercial MR equipment, however, is expensive, cumbersome, and unable to carry out non-destructive on-site measurements. In recent years, unilateral magnetic resonance (UMR) has gained popularity, due to its portability, affordability, and capacity to take non-destructive measurements [22].

**Figure 1:**
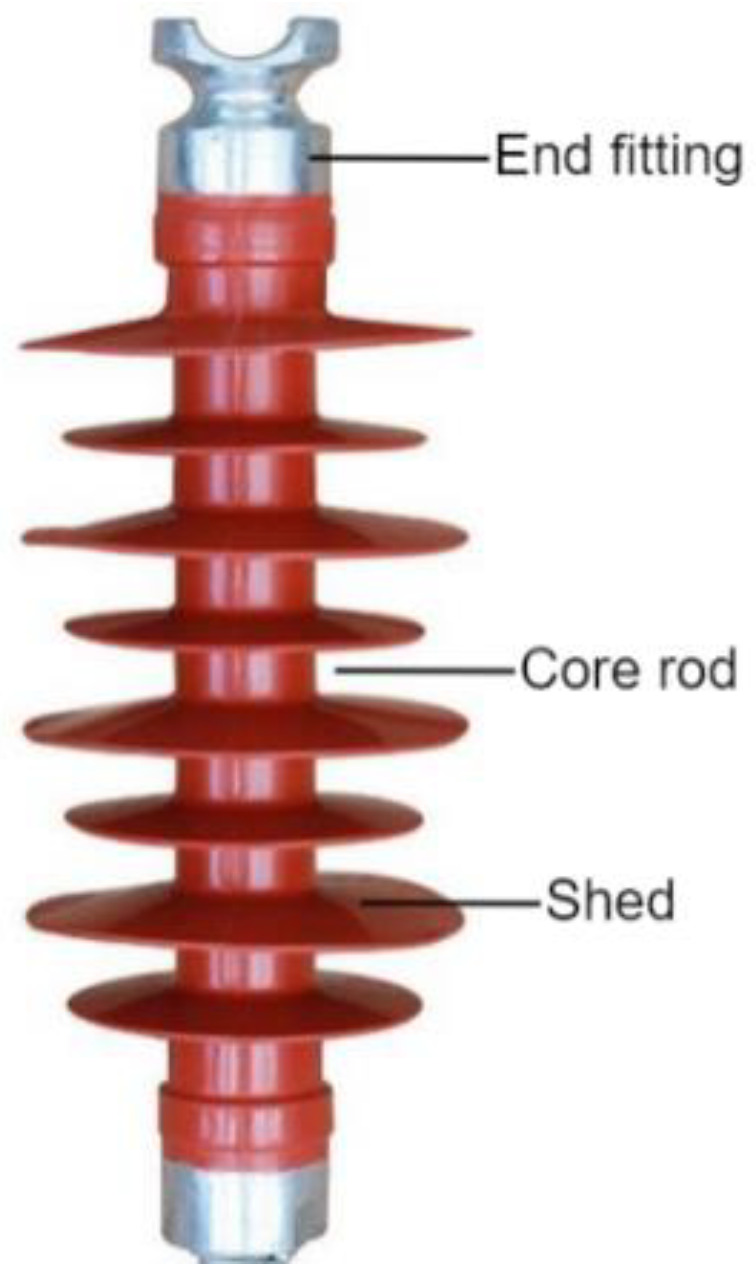
Silicone rubber insulator.

This study describes the design, fabrication, and performance testing of a portable unilateral magnetic resonance sensor employing the unilateral magnetic resonance technique. With the help of this sensor, the effective transverse relaxation times (*T*_2*eff*_) of new, 2-year-old, and 5-year-old composite insulators were measured at various depths, and on the upper and lower surfaces of the shed, respectively (Figure 1). These measurements can accurately reflect the degree of aging of SRIs. The results of the magnetic resonance measurements were also compared with those of the static contact angle method; both methods produced the same conclusions, and the magnetic resonance method was more effective at detecting alterations in distinctive parameters brought on by age.

## 2. Materials and Methods

### 2.1. Mechanism of Aging Reaction of SRI Materials

**Figure 2:**
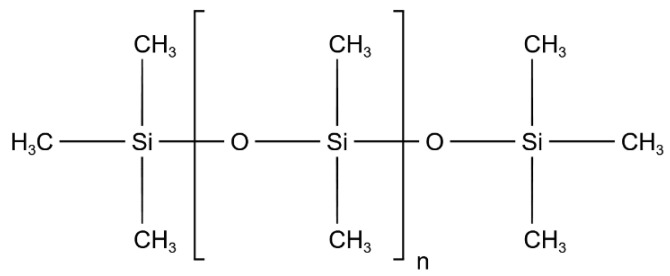
The formula of polydimethylsiloxane.

The main constituent material of SRIs is polydimethylsiloxane (PDMS), and its molecular formula is shown in Figure 2. Repeated Si-O bonds constitute the main chain of the molecule, and Si atoms are directly linked to polymers such as -CH_3_, -CH_1_, and -CH_2_. The vulcanization process strengthens the cross-linking between the major chains to create a flexible and durable polymer The strong polar effect of the Si-O bond is protected by the close arrangement of non-polar methyl groups around the Si-O backbone, which causes the silicone rubber surface to exhibit excellent hydrophobicity, and perform well as insulation [23].

According to research, when long-term severe natural circumstances such as ozone, strong UV light, and corona are combined, the Si-O link in the molecule will weaken or even break, causing the material to age irreversibly. The main chain of PDMS break, to form broken chains containing O, Si, CH_3_, and other free radicals, and cross-linking reactions between the broken chains increase the cross-link densities of SRIs [24,25]. Meanwhile, the oxidation reaction of the CH_3_ group in PDMS in the air further increases the cross-link density of the insulator surface material, which is the main reason for the embrittlement of the composite insulator surface. The cross-link density of rubber insulation has a direct correlation with transverse relaxation time. The cross-link density of the aging insulator increases, thus making its transverse relaxation time shorter. The aging status can be reflected by measuring the transverse relaxation time of composite insulator shed material [1,23,26,27,28]. The permanent magnet structure of the sensor utilized in this experiment has a far lower level of magnetic field homogeneity than a superconducting magnet. The transverse relaxation time of the sample measured under this magnetic field uniformity is habitually called the equivalent transverse relaxation time, *T*_2*eff*_.

### 2.2. Portable NMR Sensor

**Figure 3:**
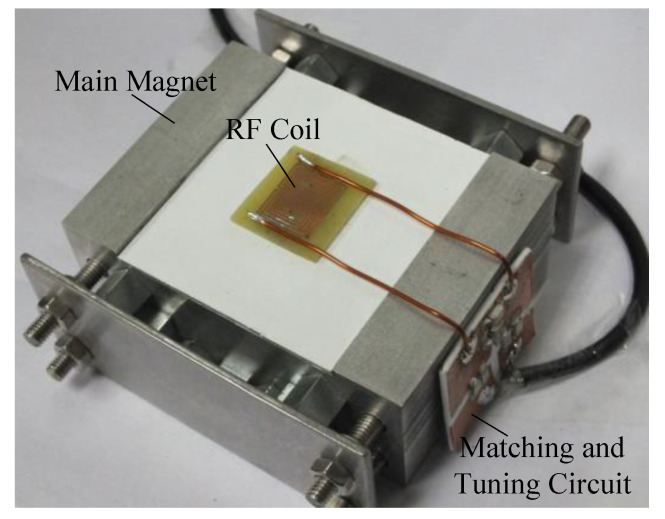
The portable NMR sensor.

In this study, according to the characteristics of the composite insulator shed, a portable NMR sensor as shown in Figure 3, was designed. The sensor consists of a main magnet, RF coil, and matching and tuning circuits (Figure 3). The main magnet is used to generate the static magnetic field, $B_{0}$, the RF coil is used to transmit the excitation signal along with the magnetic resonance signal from the sample that was received, and the matching and tuning circuit is used to achieve the impedance matching to adjust the resonant frequency to the Larmor frequency of the H atom.

**Figure 4:**
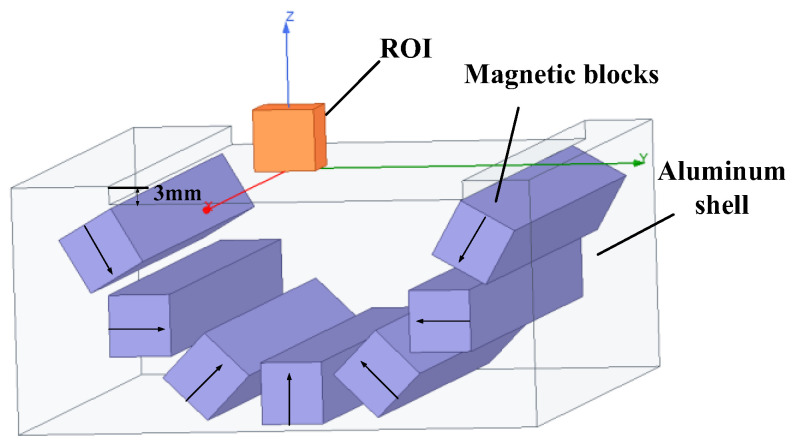
The schedule of the magnet.

The main magnet structure and coil structure are the essential design considerations for portable magnetic resonance sensors. Based on previous research work [29], this paper optimizes the Halbach [30] structure, and designed a semi-elliptical magnet structure with magnetic rods, as shown in Figure 4. The magnet establishes a static gradient magnetic field area of 10 mm × 10 mm × 4 mm above its structural surface, which is the ROI in Figure 4. The reason for establishing the static gradient magnetic field is to realize the layered depth measurement of the shed. When the sensor is attached to the surface of the shed, the material of the shed corresponds to different static-magnetic-field values in depth from the outside to the inside. According to the Larmor relationship ($\omega_{0}=\gamma B_{0}$, where $\omega_{0}$ is Larmor precession angular frequency, γ is the gyromagnetic ratio, and $B_{0}$ is the amplitude of the static main magnetic field). By adjusting the excitation frequency of the RF coil, the material at different depths on the surface and inside of the shed can be measured.

**Figure 5:**
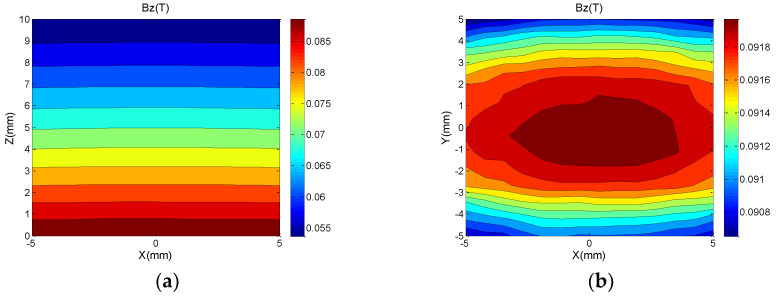
(**a**) Measured value magnetic field distribution in the area of 10 mm × 10 mm on XOZ plane y = 0 mm of ROI. (**b**) Measured value magnetic field distribution in the area of 10 mm × 10 mm on XOY plane z = 0 mm of ROI.

The arrows in Figure 4 indicate the magnetization direction of each magnetic rod. The magnetic field distribution in the optimized ROI is shown in Figure 5. In the direction perpendicular to the magnet surface, the static magnetic field decays from 96 mT on the coil surface to 80.4 mT at z = 4 mm, and the magnetic field has a flatter gradient characteristic along the z-axis, with an approximate gradient of 3.9 T/m, corresponding to an RF frequency variation of 166 kHz/mm.

**Figure 6:**
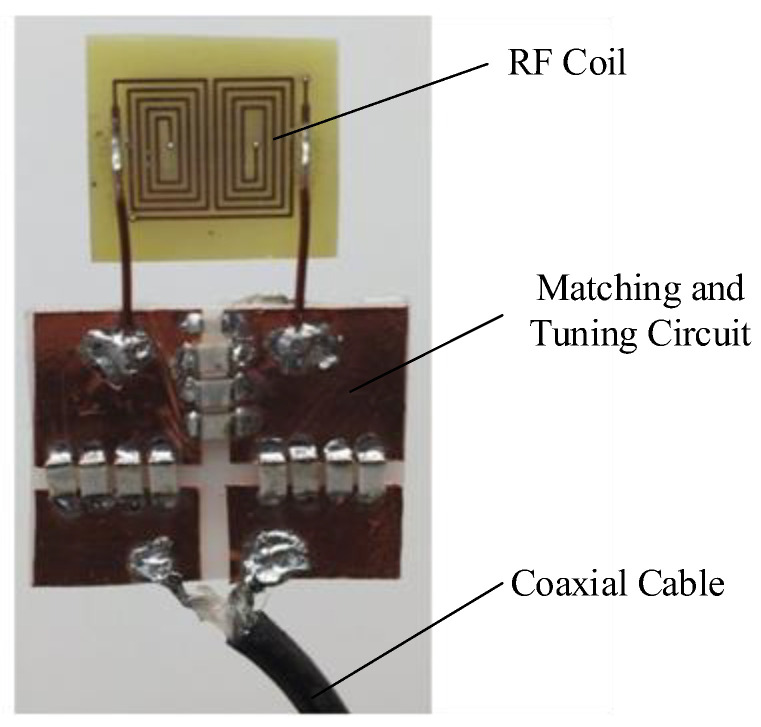
The RF coil and its matching and tuning circuit.

**Figure 7:**
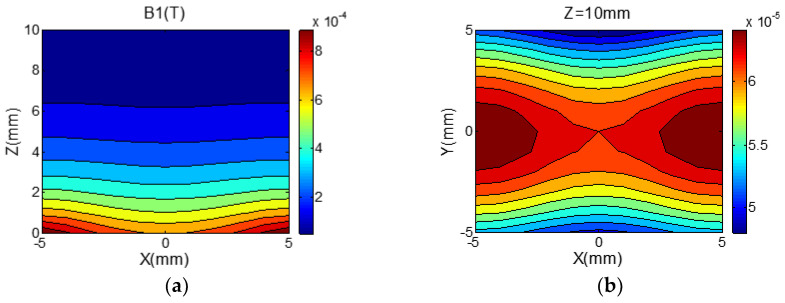
(**a**) Measured $B_{1}$ strength in ROI on transverse sections. (**b**) Measured $B_{1}$ strength in ROI on longitudinal sections.

The main magnetic field $B_{0}$ in this study is perpendicular to the magnet structure, since the condition of magnetic resonance is that it be orthogonal to the RF magnetic field. The 8-shaped coil in Figure 6 that can produce a horizontal field is chosen to make the magnetic field $B_{1}$ produced by the RF coil, perpendicular to the main magnetic field $B_{0}$. Additionally, the coil is formed of a double-layer PCB to increase the strength and uniformity of the RF magnetic field (the strength distribution of the RF magnetic field is shown in Figure 7), and the coil is made of a double-layer PCB. The coil is 22 mm long and 16 mm wide, with a total of 4.5 turns. The wire diameter is 0.5 mm, the wire spacing is 0.5 mm, the resistance is 916.5 mΩ, and the inductance is 1.15 mH.

The overall sensor size is 4.5 cm × 10.5 cm × 10.5 cm; it weighs 1.5 kg, has low production cost, and is easy to carry.

### 2.3. Experimental Method

**Figure 8:**
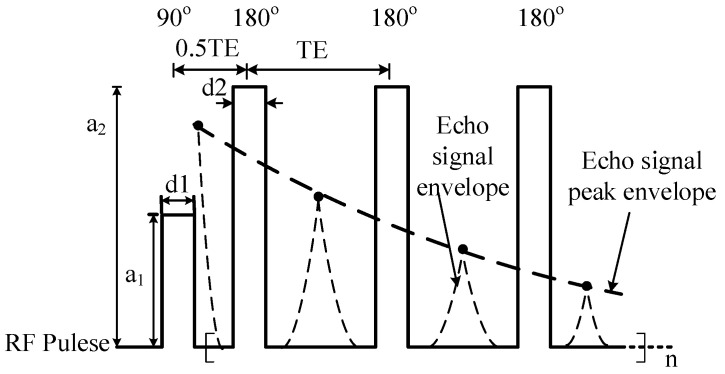
The pulse sequence of CPMG.

In this paper, the Carr–Purcell–Meiboom–Gill (CPMG) (Figure 8) RF pulse sequence [31] is used to measure the *T*_2*eff*_ value, and the parameters of the CPMG sequence are set as shown in Table 1. In Figure 8, the peak point of the spin-echo signal between two 180° RF pulses is connected to obtain the peak envelope line of the spin-echo signal, and the *T*_2*eff*_ value can be obtained by Laplace inversion of this line.

In [1,27,32], it has been verified that solids and liquids have different transverse relaxation times. In this study, the sensor was used to test two different types of samples in the liquid–solid state and delamination model, respectively, to confirm the sensor’s accuracy in measuring the equivalent transverse relaxation time and its capacity to perform delamination measurements.

**Figure 9:**
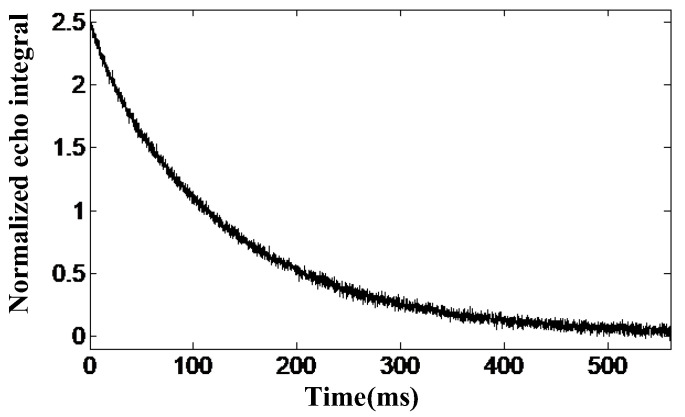
CPMG echoes the envelope curve of pure water.

The two samples in the liquid–solid state were water and rubber, and the CPMG echo envelope obtained from the water sample test is shown in Figure 9. The *T*_2*eff*_ value of pure water was 131.4 ms after fitting the curve to a single exponential. Under the same test conditions, the *T*_2*eff*_ value of rubber was 12.1 ms. The results of the experiments demonstrate that the corresponding transverse relaxation time decays for liquids significantly more slowly than for solids, which initially validates the reliability of the sensor for measuring the *T*_2*eff*_ value of the sample.

**Figure 10:**
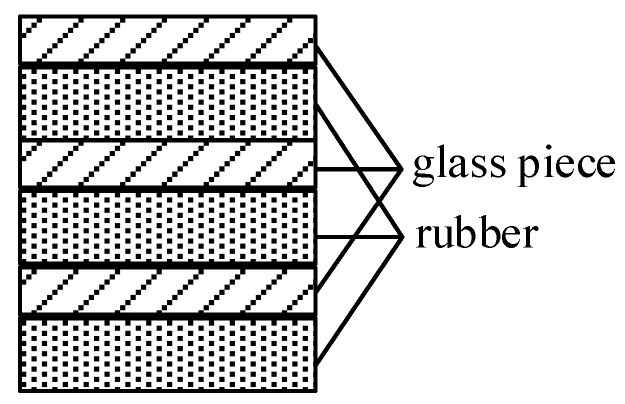
Illustration of layered samples.

The main magnet of this sensor has a constant gradient of 3.9 T/m in the vertical direction, and its corresponding frequency variation is 166 kHz/mm. The one-dimensional layered-model identification experiment uses three layers of samples (separated from each other by a glass sheet) that are put onto the surface of the RF coil and excited with CPMG pulses. The position of the measured sample is reflected by the measured wave-peak position. Three pieces of rubber of 10 mm × 10 mm × 0.2 mm and several pieces of glass of 140 μm were used as the samples for this experiment, and the layering schematic is shown in Figure 10. The excitation frequency is the frequency of the main magnet magnetic field corresponding to the middle position of the total sample, which is 3.98 MHz, the pulse width is 2 μs, the TE is 210 μs, and the sampling point is 64. Since the intensity of the echo signal received in the experiment varies with time, the signal can be transformed from the time domain to the frequency domain by the Fourier transform, to obtain the frequency range of the signal.

**Figure 11:**
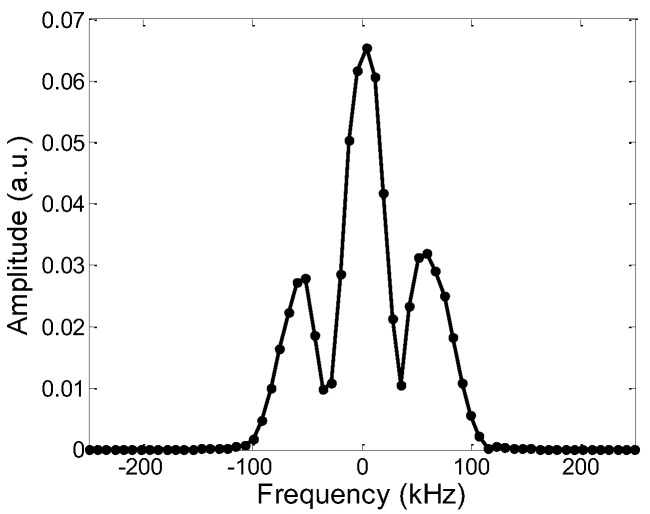
The result of identifying layered samples by the FFT method.

Figure 11 shows the frequency distribution of the measured sample. The frequency difference between the two adjacent peaks is 54.875 kHz and 57.0781 kHz, corresponding to a thickness of 331 μm and 344 μm, respectively, with a maximum deviation of 2.6% from the true value of 340 μm. The experiment proves that the sensor can distinguish the sample distribution more accurately in the height direction. In conclusion, the magnetic resonance sensor can precisely determine the sample distribution in the height direction, and quantify the effective transverse relaxation time of solids and liquids.

In this paper, three types of insulators are tested and studied. One brand-new composite insulator (sample A), and the other two have been used in the field for nearly 2 years (sample B) and 5 years (sample C). Since the research in this paper is a non-destructive testing method, there is no need to destroy the insulator shed, and the shed to be tested only needs to be cleaned with water and dried naturally during pretreatment. The way of hanging the insulator may cause different aging and damage conditions on the upper and lower surfaces of the same shed. To detect this change, the upper and lower surfaces of the same shed are regarded as different samples. The number of each sample is shown in Table 2.

**Figure 12:**
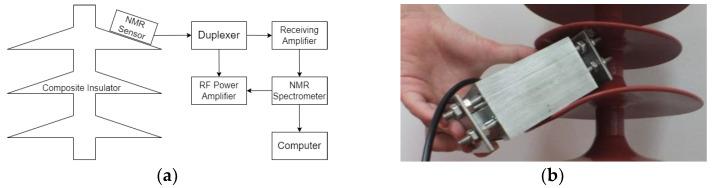
(**a**) Diagram of the sensor measurement principle; (**b**) diagram of the sensor measurement method.

The overall measurement system structure is shown in Figure 12a, and the field measurement is shown in Figure 12b. The magnetic resonance sensor is attached to the surface of the insulator sheds for measurement.

**Figure 13:**
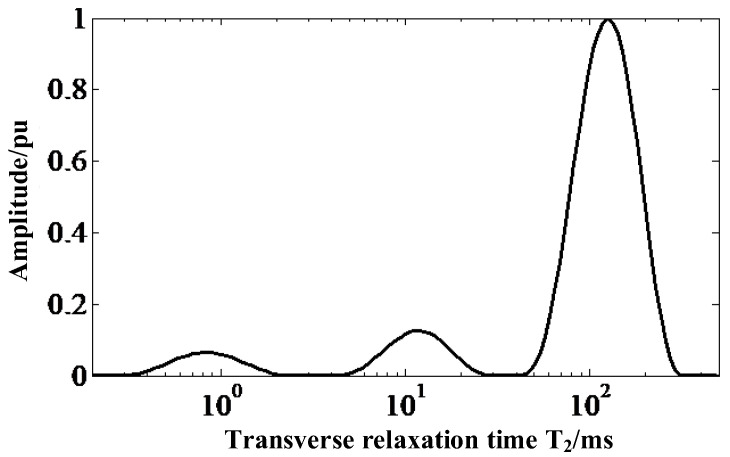
*T*_2_ distribution by Inverse Laplace Transform.

It can be seen from the molecular formula of the SRIs that the H atom is present in more than one group in its molecular formula, so its transverse relaxation time is not single. After the CPMG echo envelope of the sample is measured, the distribution of its transverse relaxation time is found not by single exponential fitting but by inverse Laplace transform inversion (shown in Figure 13). Finally, the equivalent transverse relaxation time, *T*_2*eff*_ of the sample can be extracted by Equation (1).

(1) $$T_{2eff}=\int_{T_{2-min}}^{T_{2-max}}f\left(T_{2}\right)T_{2}dT_{2}/\int_{T_{2-min}}^{T_{2-max}}f\left(T_{2}\right)dT_{2}$$

In Equation (1), $T_{2min}$ and $T_{2max}$ represent the start time and end time of each wave of the curve shown in Figure 13, and $f\left(T_{2}\right)$ is the *T*_2_ distribution curve function.

## 3. Experiments and Results

### 3.1. T_2eff_ Measurement of Insulators with Different Operating Times

Firstly, the samples were observed visually. The surface of the new insulator A sample was bright red, sample B with 2 years of online operation was dark red, and the surface of sample C with 5 years of online operation had a certain degree of hardening and chalking, and the color was basically light red. To ensure the comparability of the experimental data, all experiments were repeated three times, and *T*_2*eff*_ was the average of the three measurements of the sample, with the same experimental parameters and the same treatment methods in the same group. In this experiment, the same measurement depth (1.4 mm from the upper surface) was maintained, and the *T*_2*eff*_ values of samples with different operation times were measured, with an excitation frequency of 3.86 MHz.

**Figure 14:**
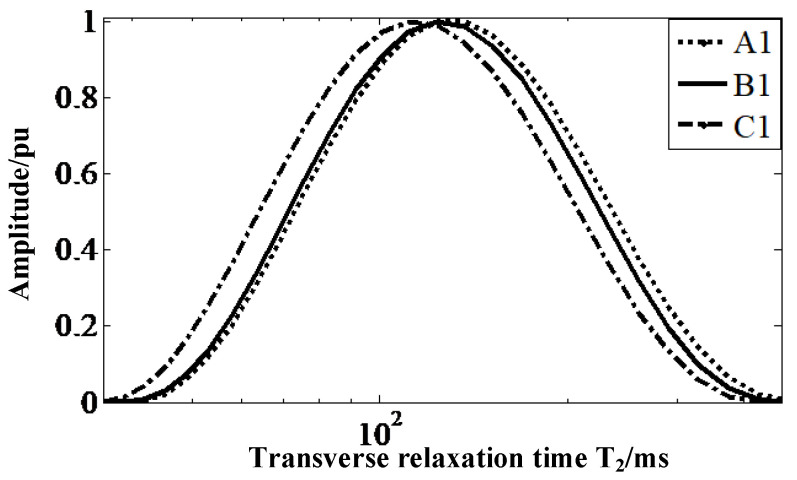
*T*_2_ distribution of the samples.

Figure 14 shows the distribution of transverse-relaxation-time spectra on the upper surface of the three insulators. The dotted (C1) and solid (B1) lines gradually shift to the left, compared with the dashed (A1) lines, marking the fact that the *T*_2*eff*_ of the insulators gradually decreases as their service life increases. The *T*_2*eff*_ of the three insulators are shown in Table 3, and the comparison reveals that the *T*_2*eff*_ values of the new insulators are 4.47% higher than those of the insulators with 2 years of operation, and approximately 19.31% higher than those of the insulators with 5 years of operation

### 3.2. Measurement of Difference between Upper and Lower Surfaces of the Same Shed

**Figure 15:**
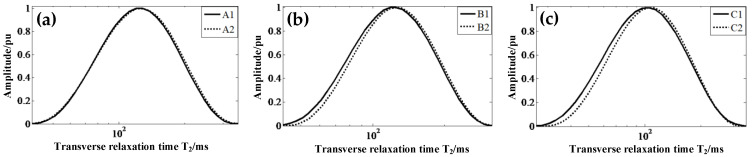
(**a**) One-dimensional spectral curves of transverse relaxation times of A1 and A2 samples. (**b**) One-dimensional spectral curves of transverse relaxation times of B1 and B2 samples. (**c**) One-dimensional spectral curves of transverse relaxation times of C1 and C2 samples.

The shape and usage of composite insulators determine that the aging of the same shed’s upper and lower surfaces may differ. In this experiment, the *T*_2*eff*_ values of the upper and lower surfaces of three insulator samples with different running times are measured. The excitation frequency is 4.08 MHz. The *T*_2_ spectrum distribution of each sample is shown in Figure 15.

As seen in Figure 15, the solid line (upper surface) shifts to the left, compared with the dotted line (lower surface), and the trend of the left-shift is gradually clear from Figure 15a–c. It indicates that the upper and lower surfaces of the insulators age differently, and that this difference becomes gradually more obvious as service time increases. The *T*_2*eff*_ of the upper and lower surfaces of different samples are shown in Table 4. After comparison, the *T*_2*eff*_ value of the lower surface of the new insulator changes by approximately 0.93% compared with the upper surface, which is within the measurement error of the sensor, and it is difficult to distinguish the upper and lower surfaces of the insulator by data comparison. The *T*_2*eff*_ values of the lower surface of insulators that have been in operation for 2 and 5 years are approximately 3% and 5.08% higher than those of the upper surface, respectively, and it is basically possible to distinguish the upper and lower surfaces from the numerical values. Therefore, the mobile MRI measurement results can reflect the difference in the aging degree between the upper and lower surfaces of the composite insulator shed; the *T*_2*eff*_ values on the upper surface of the aging insulator are smaller than those on the lower surface, and the larger the difference between the *T*_2*eff*_ values on the upper and lower surfaces, the more serious the aging.

### 3.3. Insulator Delamination Measurement

**Figure 16:**
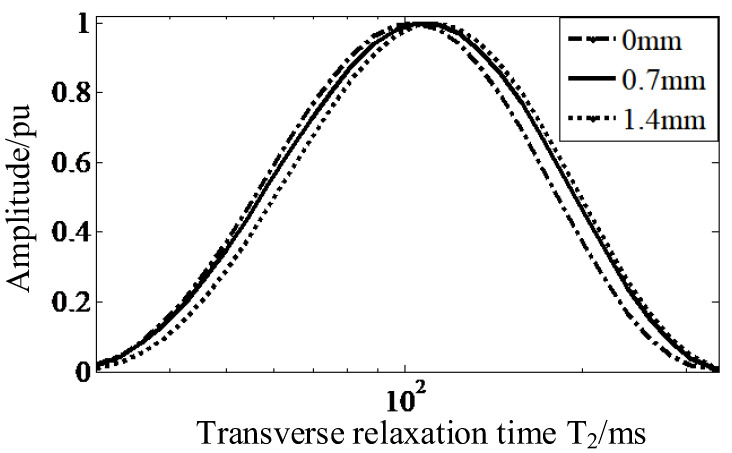
The *T*_2_ distribution of C1 at different depths.

The surface material of the shed is inevitably more severely aged than its internal material. To distinguish this phenomenon of layered aging, the equivalent transverse relaxation time at different depths was measured on the upper surface of the insulator with 5 years of service, in this round of experiments. The measurement frequencies were set to 4.08 MHz, 3.97 MHz, and 3.86 MHz, and the corresponding measurement depths were 0 mm, 0.7 mm, and 1.4 mm, respectively. Figure 16 shows the distribution spectrum of transverse relaxation times measured at different depths of the samples.

The *T*_2_ spectrum distribution curves of C1 samples were gradually shifted to the right from the depth of 0 mm through 0.7 mm to 1.4 mm, and the *T*_2*eff*_ at different depths are shown in Table 5. Comparing their *T*_2*eff*_ values, it is found that the *T*_2*eff*_ value at 1.4 mm is the largest, which is 3.51% higher than that at 0.7 mm and 9.46% higher than that at 0 mm. It can be seen from the changing trend that the aging of the shed material gradually slows down, and the *T*_2*eff*_ value gradually increases with the increment of the measurement depth.

### 3.4. Comparison with Static Contact Angle Method Test Results

**Figure 17:**
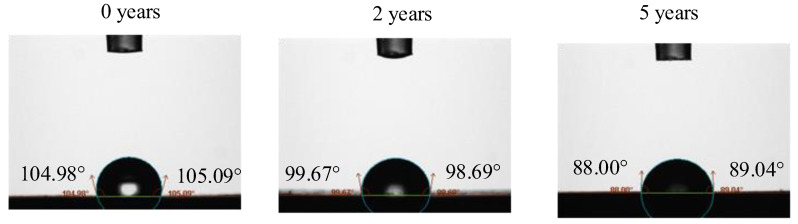
The static contact angle of the samples after 10 min.

**Figure 18:**
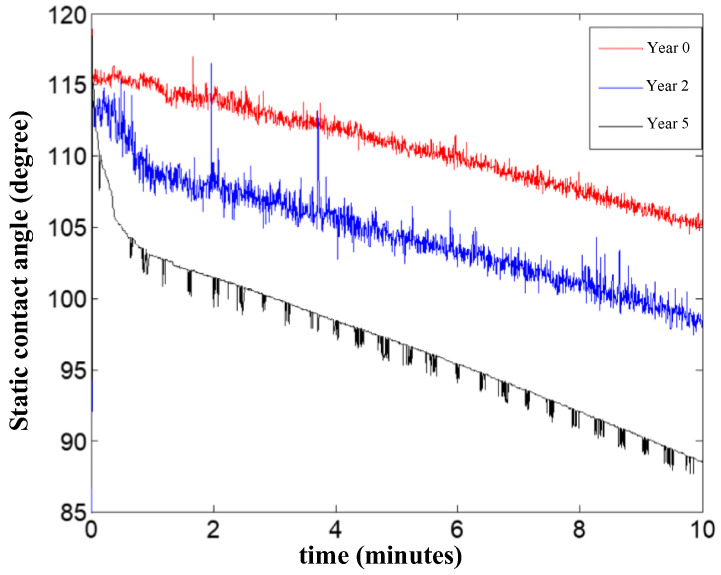
Static contact angle with a testing time of the samples.

To verify the measurement results of the above magnetic resonance method, the same samples were also measured using the static contact angle method with a THETA series contact angle measuring instrument from Attension, Sweden. The instrument automatically measured static contact angles at room temperature with a volume of 3.5 μL of water drops on the sample surface for 10 min. The instrument measured the left and right static contact angles automatically, and the static contact angle results at the 10th min are shown in Figure 17. The average of the two values at each time point was taken as the static contact angle value at that time, and the results of the static contact angle of the three samples with time are shown in Figure 18.

From the measurement results in Figure 18, with the extension of the measurement time, the water droplets on the solid surface evaporate continuously, and the droplet volume shrinkage becomes smaller; at the same time, due to the difference in the surface energy of the sample to be measured, the droplet spreads to different degrees, which means the contact angle tends to decrease. To reduce the influence of water evaporation, the average value of the three-time points of 9.5 min, 10 min, and 10.5 min (as shown in Figure 17, each time point measured the left and right two static-contact-angle values) was selected as the static-contact-angle value of the insulator sample, and the test results are shown in Table 6.

**Figure 19:**
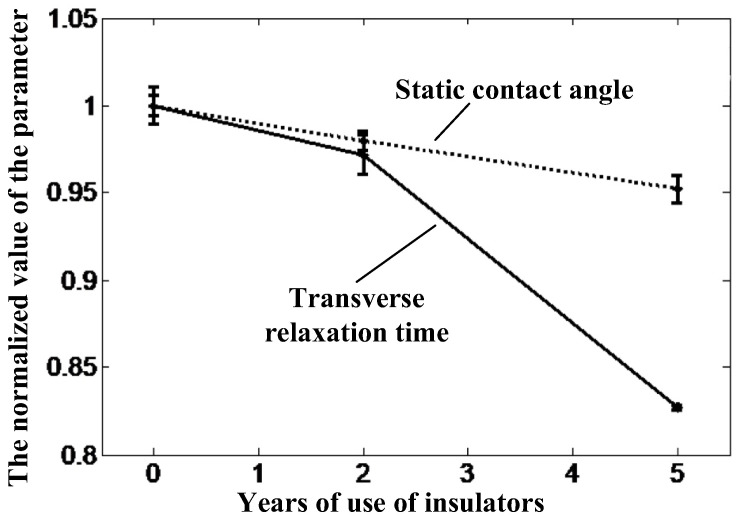
*T*_2*eff*_ and static contact angle of the insulators.

Figure 19 shows the normalized trends of *T*_2*eff*_ and static-contact-angle-values on the upper surface of the three insulators, relative to the new insulator data. It can be seen that the trends of the data measured by the static contact angle method and the magnetic resonance method are the same. With the increase of the aging degree, both the static contact angle and *T*_2*eff*_ show a decreasing trend, and the change of *T*_2*eff*_ is more obvious, which makes it easier to distinguish the aging status of different insulators.

## 4. Conclusions

In this paper, a portable magnetic resonance non-destructive testing method is proposed for the aging of SRI materials. The method is not only able to measure the aging of the surface of SRI materials, but also to detect the aging of their internal conditions. From the experimental results, it can be concluded that: (1) the longer the composite insulator is in service, the more severe the aging, and that its corresponding *T*_2*eff*_ decreases; (2) the insulator sheds from the surface to the inside, *T*_2*eff*_ gradually increases, and the aging state phenomenon gradually is reduced; (3) compared with the static contact angle method, the characteristic parameters of the magnetic resonance measurement method change more obviously with the aging of the material. This study is of significance for a better understanding of the aging law of SRIs in the longitudinal direction, a scientific assessment of their service life, and a reasonable evaluation of their quality. 

Although this study initially demonstrated the feasibility of this new method, it is limited by the number of samples that can be tested. More samples need to be selected and more data need to be collected, to derive a rule for assessing the aging status of composite insulators.

## Figures and Tables

**Table 1:** Parameters of CPMG sequence.

PW	TE	ATT of 90°	NES	TR	NS
4 μs	140 μs	−18 dB	1000	400 ms	128

**Table 2:** Sample number of all sheds.

Sample	Upper Surface	Lower Surface
Sample A	A1	A2
Sample B	B1	B2
Sample C	C1	C2

**Table 3:** *T*_2*eff*_ of the samples.

Sample	*T*_2*eff*_ (ms)	Standard Deviation
A1	141.3	0.6
B1	135.0	0.9
C1	114.0	2.4

**Table 4:** Values of *T*_2*eff*_ of the samples on the upper and lower surfaces.

Sample	*T*_2*eff*_ (ms)	Standard Deviation
A1	130.3	1.4
A2	131.5	0.6
B1	126.6	1.5
B2	130.5	1.2
C1	107.8	0.2
C2	113.5	0.5

**Table 5:** Values of *T*_2*eff*_ in the aging test at different depths.

Measurement Depth(mm)	*T*_2*eff*_ (ms)	Standard Deviation
0	107.8	0.2
0.7	114.9	0.4
1.4	119.0	2.1

**Table 6:** Values of static contact angle of different silicone rubber insulators.

Sample	Static Contact Angle (Degree)	Standard Deviation
A1	115.27	0.67
B1	112.94	0.66
C1	109.76	0.93

## Data Availability

Not applicable.
